# KinMap: a web-based tool for interactive navigation through human kinome data

**DOI:** 10.1186/s12859-016-1433-7

**Published:** 2017-01-05

**Authors:** Sameh Eid, Samo Turk, Andrea Volkamer, Friedrich Rippmann, Simone Fulle

**Affiliations:** 1 BioMed X Innovation Center, Im Neuenheimer Feld 515, 69120 Heidelberg, Germany; 2 Computational Chemistry and Biology, Merck KGaA, Frankfurter Str. 250, 64293 Darmstadt, Germany

**Keywords:** Protein kinases, Human kinome tree, Interactive annotation, Images

## Abstract

**Background:**

Annotations of the phylogenetic tree of the human kinome is an intuitive way to visualize compound profiling data, structural features of kinases or functional relationships within this important class of proteins. The increasing volume and complexity of kinase-related data underlines the need for a tool that enables complex queries pertaining to kinase disease involvement and potential therapeutic uses of kinase inhibitors.

**Results:**

Here, we present KinMap, a user-friendly online tool that facilitates the interactive navigation through kinase knowledge by linking biochemical, structural, and disease association data to the human kinome tree. To this end, preprocessed data from freely-available sources, such as ChEMBL, the Protein Data Bank, and the Center for Therapeutic Target Validation platform are integrated into KinMap and can easily be complemented by proprietary data. The value of KinMap will be exemplarily demonstrated for uncovering new therapeutic indications of known kinase inhibitors and for prioritizing kinases for drug development efforts.

**Conclusion:**

KinMap represents a new generation of kinome tree viewers which facilitates interactive exploration of the human kinome. KinMap enables generation of high-quality annotated images of the human kinome tree as well as exchange of kinome-related data in scientific communications. Furthermore, KinMap supports multiple input and output formats and recognizes alternative kinase names and links them to a unified naming scheme, which makes it a useful tool across different disciplines and applications. A web-service of KinMap is freely available at http://www.kinhub.org/kinmap/.

**Electronic supplementary material:**

The online version of this article (doi:10.1186/s12859-016-1433-7) contains supplementary material, which is available to authorized users.

## Background

Protein kinases are key effectors in the intracellular signal transduction pathways and, when dysregulated by mutations or overexpression, can cause the progression of diseases such as cancer and inflammation [1]. Since the clinical success of Gleevec (imatinib) in the treatment of chronic myeloid leukemia [2], protein kinases have become among the most pursued drug targets for cancer. The human kinome comprises nearly 540 kinases which were initially classified by Manning et al. based on the underlying sequences into eight typical groups (AGC, CAMK, CK1, CMGC, STE, TK, TKL, Other) and 13 atypical families [3]. The resulting phylogenetic tree is commonly used to visualize compound profiling data [4, 5] or structural features of kinases [6, 7]. A continuously growing body of knowledge is available, covering not only structural and biochemical aspects but also data related to diseases and genetic modifications. Hence, a tool that integrates kinome-related data from multiple resources would allow exploration of complex queries pertaining to kinase involvement in the pathophysiology of various disorders as well as to the disease-modulating potential of protein kinase inhibitors. To date, a few kinome tree viewers have been developed to facilitate visualization tasks such as the TREEspot tool from DiscoveRx [8], the NCGC Kinome Viewer [9], and Kinome Render [10]. The former two were primarily designed for the visualization of compound profiling data but they do not allow the annotation of further information in a straightforward manner. Kinome Render offers a wider variety of annotation formats and customizable text labels, but it requires a specialized input file format and does not accept input from commonly used formats such as spreadsheets. Moreover, Kinome Render only creates static images and does not allow interactive linking to other kinome-related resources. The mentioned limitations of already existing kinome tree viewers motivated us to develop KinMap.

**Fig. 1:**
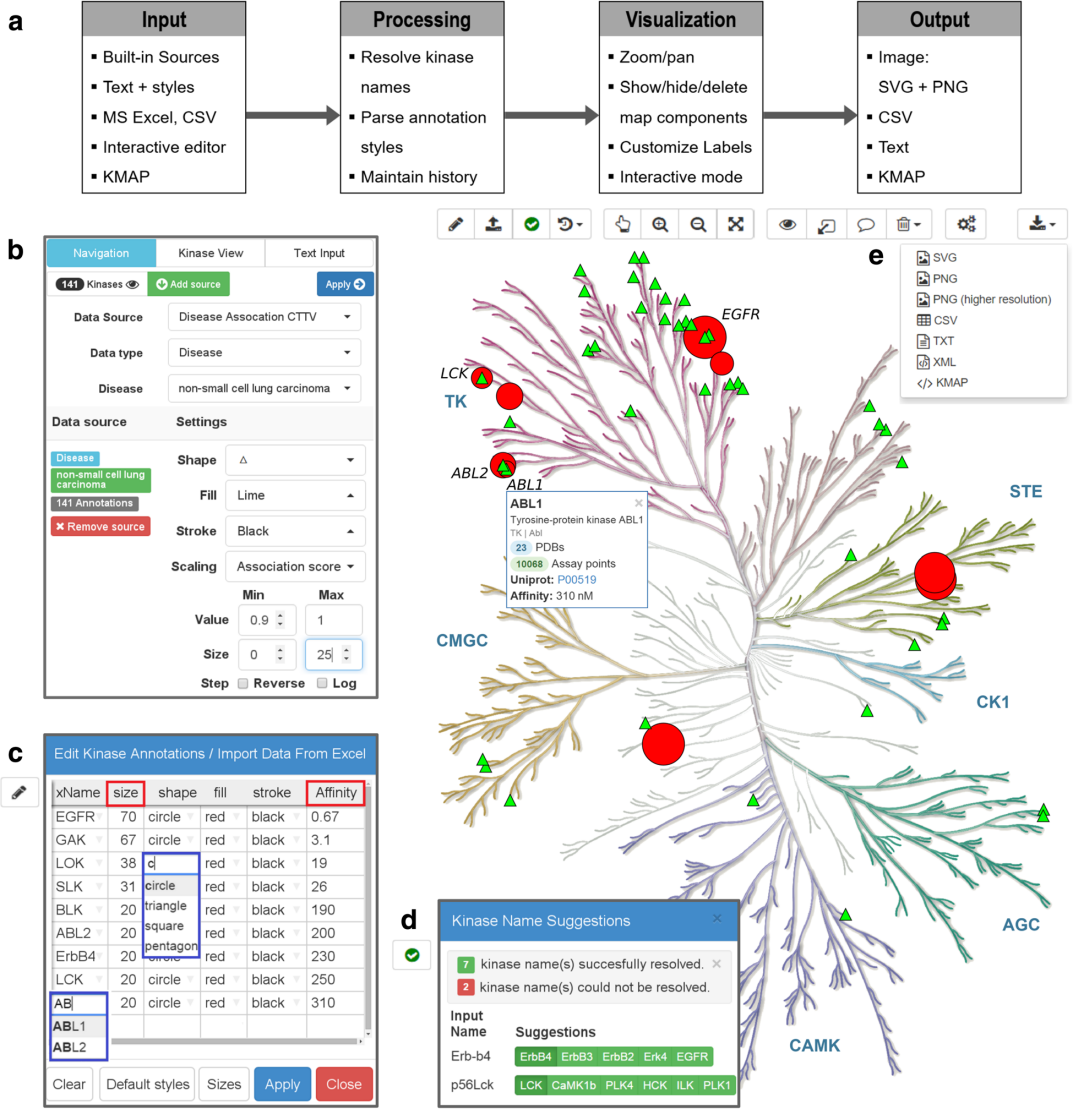
The KinMap web interface. **a** Summary of the key features. **b** Navigation panel which allows linking kinome related data sources, e.g. non-small cell lung carcinoma from CTTV (*green triangles*). **c** Preview of the built-in spreadsheet editor showing inhibition profile of erlotinib (*red circles* on the tree) [4]; auto-complete functions (*blue rectangles*) facilitate kinase selection and style modification. Annotation sizes can be automatically rescaled based on bioactivity data (*red rectangles*) or other input values in the spreadsheet. **d** Kinase name suggestions in case of incomplete or ambiguous names in the input. **e** Supported output formats CSV: comma-separated values; PNG: Portable Network Graphics; SVG: Scalable Vector Graphics; XML: extensible markup language; KMAP: native KinMap format. A box showing detailed information for the ABL1 kinase illustrates the interactive view mode

KinMapis a web-based tool for creating interactive annotations of the phylogenetic tree of the human kinome and facilitating navigation through kinase-related data such as the number of available PDB structures, number of compound data in ChEMBL, inhibitor profiling data sets, kinase disease associations, as well as proprietary data. The online tool supports various input and output formats (e.g. spreadsheets), recognizes alternative kinase naming schemes and produces high-resolution images. Finally, KinMap employs modern web technologies to run entirely locally in the web browser without uploading sensitive data to a server. The key features of KinMap are summarized in Fig. 1a and discussed in detail below.

## Implementation

### Kinome navigation via precompiled data sources

To facilitate the exploration of kinase-related information, structural, biochemical, and functional data were extracted from external resources and integrated into KinMap. Briefly: the number of kinase structures were obtained from the Protein Data Bank [11], the number of assay and compound values from ChEMBL [12], inhibition profiles from published panels [4, 13], and information related to kinase-disease associations from the Center for Therapeutic Target Validation (CTTV) platform [14]. The current version of these data sources can be found online at http://www.kinhub/kinmap/help-sources.html. In the case of the CTTV data, entries with low association scores (<0.1) were excluded, while only disease associations linked to one or more of the following evidence types were kept: genetic, somatic mutations, drugs, affected pathways or RNA expression [14]. Eventually, this allows the user to visually investigate kinase involvement in more than 670 diseases across 16 therapeutic areas.

A specialized *navigation mode* in KinMap allows interactive exploration of the relationships between these data sources. Annotations can be added to the tree directly from the built-in data sources, individually styled, and interactively resized using relevant metrics such as inhibition constants. Figure 1 outlines the key features of KinMap and illustrates the combined annotations of the inhibition profile of erlotinib [4] and kinases associated with non-small cell lung carcinoma [14]. The annotated tree in Fig. 1 exemplifies the identification of key targets, e.g. EGFR and ABL1, responsible for the therapeutic action of erlotinib.

### Data input formats

In addition to the precompiled data sources, users can create customized annotations in KinMap by using various input formats ranging from a simple list of kinases to complex spreadsheets and by adjusting the styles, sizes, and text labels on the fly. KinMap features an interactive spreadsheet editor which enables the user to import annotations from spreadsheets, to add or delete annotations, and to modify annotations styles and sizes. Drop-down menus with auto-complete functions increase the convenience of adjusting annotations in the spreadsheet editor. Moreover, KinMap can read additional data from CSV input files, e.g. bioactivity values, whereby the user can readjust annotation sizes using the automated rescaling function in the spreadsheet editor (Fig. 1c). Additional data can also serve as information sources for the interactive kinome view (discussed below). Finally, KinMap supports a minimalistic text input for less sophisticated annotations. For example, the following concise syntax annotates eight kinases potentially involved in cardiomyopathy [15]:

The first line defines the annotation style of the succeeding kinases until another assignment is specified. In the present example, kinases are marked as squares that are 25 × 25 pixels in size, colored in red, and surrounded by a grey border.

### Kinase names

Different naming schemes and abbreviations of kinases are used by researchers across different groups and/or disciplines. To account for this, KinMap contains a versatile parsing function that links commonly used kinase names to a unified naming scheme. The current parsing function recognizes names used by Manning [3], recommended by the UniProt Consortium [16], approved by the HUGO Gene Nomenclature Committee [17], as well as any of the alternative names listed in these resources. Moreover, the parsing function accepts incomplete or ambiguous kinase names and allows the user to select the intended kinase from a prioritized list of potential matches in a specialized interface (Fig. 1d).

### Visualization and interactive kinome view

KinMap can be used to produce sophisticated kinome tree annotations integrating information from multiple sources. The increasing availability and complexity of kinase-related information can easily result in cluttered kinome tree annotations that might be hard to grasp. Therefore, we implemented additional visualization options in KinMap to maximize the usability of the annotated kinome tree, e.g. zoom and drag the view, customize text labels, and toggle tree components on and off. In addition, KinMap introduces an interactive mode providing a hovering function which can display detailed information for a particular kinase such as inhibition assay results (e.g. ABL1 in Fig. 1). The interactive view is particularly useful for browsing and sharing complex data without overloading the information content of the annotated kinome tree.

### Output formats and data exchange

KinMap allows exporting annotated maps to *static* high-quality images (PNG and SVG), e.g. for use in journal publications and posters. Additionally, KinMap supports three *editable* file formats: plain text (TXT), comma-separated values (CSV), extensible markup language (XML), and native KinMap (KMAP) format. The minimalistic plain text format (see example above) saves lists of kinases and concise style directives making it suitable for highlighting distinct subsets of kinases by different annotation styles. The CSV format is more expansive and can be modified using text editors or spreadsheet software, e.g. Microsoft Excel, to facilitate data exchange. Finally, the native KMAP format preserves the metadata required to exactly reproduce the view of KinMap, e.g. label font settings, zoom level, and interactive mode settings.

## Results and discussion

**Fig. 2:**
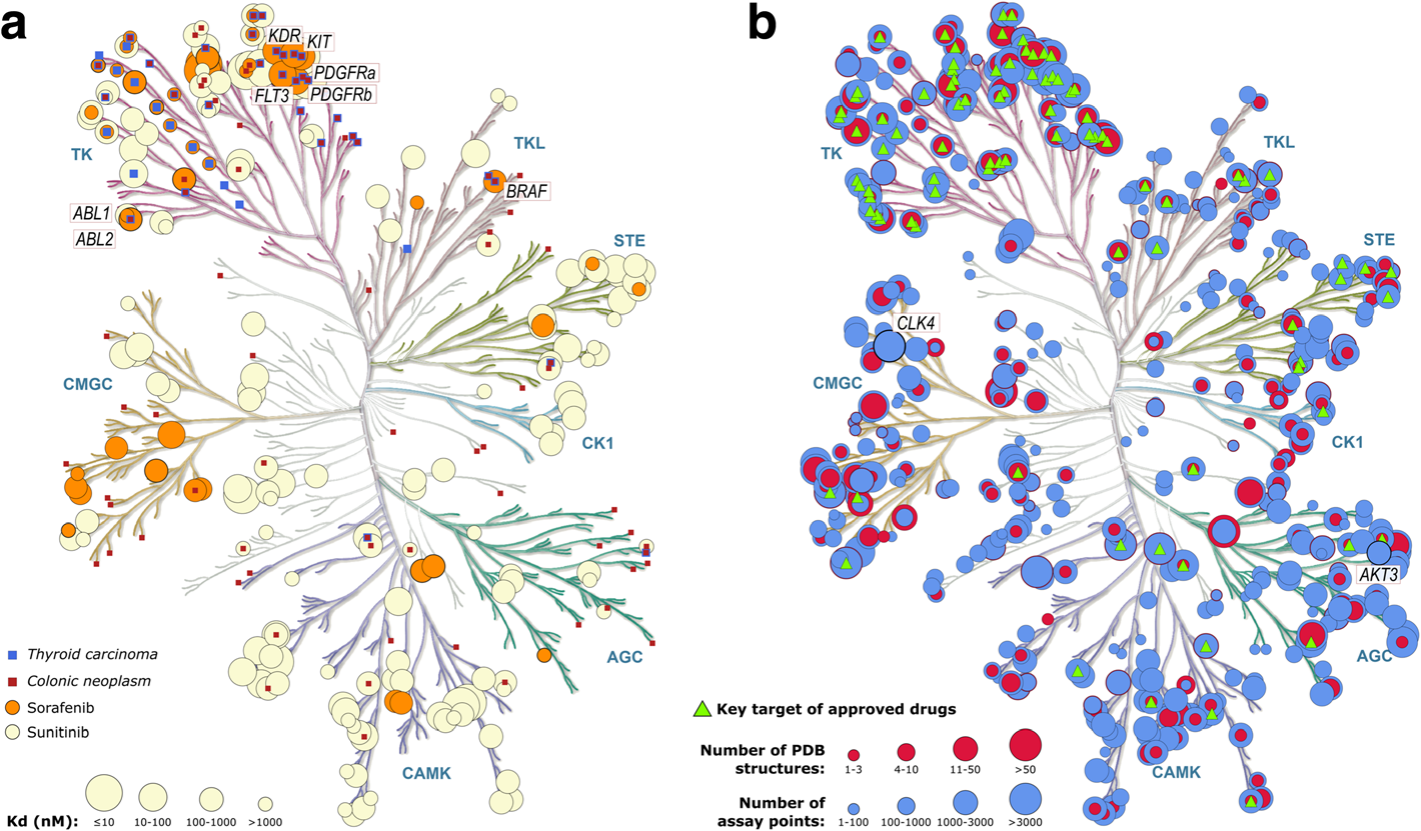
Examples of kinome tree annotation created by KinMap. **a** Inhibition profiles of two approved drugs sorafenib (*orange circles*) and sunitinib (*creme circles*) [4], and kinases linked to thyroid carcinoma (*blue squares*) and colonic neoplasm (*red squares*) as extracted from the CTTV platform [14]. **b** Availability of structural and bioactivity data for the human kinome; *red circles*: number of structures per kinase in the PDB [11]; *blue circles*: number of activity measurements for the respective kinase in ChEMBL [12]; *green triangles*: key targets of clinically approved kinase inhibitors [12]

KinMap facilitates visualization of data from different resources such as structural, biochemical, and functional data, and allows not only generation of high-quality pictures but also interactive exploration of connections. The navigation possibilities will be now exemplarily demonstrated in two test cases (Fig. 2). The first one aims to identify new therapeutic applications for known kinase inhibitors and the second one to prioritize kinases for drug development projects. Instructions to produce these illustrations are available in online tutorials at http://www.kinhub/kinmap/tutorial.html and the corresponding input files are provided in the Additional file 1.

### Exploring novel therapeutic indications

Over the past few decades the development costs for new drugs has increased dramatically, but this was paradoxically accompanied by a decline in the likelihood of approval in the clinical testing phases [18]. However, clinically approved drugs and investigational drugs that fail due to lack of efficacy or economic reasons could still be leveraged to find new indications, a process referred to as drug repurposing. This approach is economically appealing as these drugs have already passed the expensive early phases of clinical testing and have demonstrated good safety profiles and, therefore, have lower chances of subsequent clinical failure [19]. Exploring novel indications is particularly valuable in the case of anti-neoplastic kinase inhibitors due to the lower phase 1 success rate of oncology drugs (6.7%) compared to other indications [18].

Figure 2a shows the inhibition profile of two clinically approved kinase inhibitors, sorafenib and sunitinib, against a panel of 317 protein kinases [4]. Sorafenib inhibits several tyrosine protein kinases, such as VEGFR, PDGFR and Raf family kinases. It was initially approved by the FDA for the treatment of patients with advanced renal cancer in 2005 [20], and later also for hepatocellular carcinoma [21]. Sunitinib inhibits a number of receptor tyrosine protein kinases including PDGFR (alpha and beta), VEGFR2 (KDR), KIT, FLT3 and RET, which are key factors in tumorigenesis and neoplastic cell proliferation [22]. In 2006, sunitinib was approved by the FDA as a treatment for gastrointestinal stromal tumors and renal carcinoma as well as for a rare type of pancreatic cancer in 2011. Additionally, Fig. 2a shows the kinases associated with two types of cancers for which both drugs have not been initially approved: thyroid and colon carcinomas [14]. The overlap between several key targets of sorafenib and sunitinib, and kinases implicated in thyroid and colon carcinomas indicates that the two compounds are promising candidates for treatment of both cancer types. Interestingly, a 2010 study of off-label use of both drugs demonstrated the efficacy in patients with widely metastatic, progressive differentiated thyroid cancer [23]. Subsequently, sorafenib was approved by the FDA for the treatment of metastatic differentiated thyroid cancer in 2013, while sunitinib is currently in phase 2 clinical trial as an adjunctive treatment for advanced differentiated thyroid cancer [24]. Moreover, off-label use of sorafenib showed positive results in personalized colon cancer therapy in a number of cases: e.g. combining sorafenib with cetuximab and panitumumab resulted in a notably long period of progression-free survival in a patient with V600E BRAF-mutant colon cancer [25]. Furthermore, a metastatic colorectal cancer patient with FLT3 mutation showed significant symptomatic and laboratory improvement with sorafenib treatment [26]. In line with these examples, KinMap facilitates combining biochemical and disease pathology information which can help uncover potential therapeutic indications of known kinase inhibitors.

### Structure and bioactivity data

Current structural and biochemical coverage of the human kinome as well as the distribution of primary targets of clinically approved inhibitors are shown in Fig. 2b, revealing some interesting insights. Firstly, most of the kinases targeted by approved drugs (green triangles) are located in the TK and TKL groups, leaving ample therapeutic opportunities for drug development against clinically relevant kinases in other groups. Secondly, nearly 200 kinases lack any experimental structures (red circles) in the PDB [11] despite having abundant biochemical assay results (blue circles). For instance, the dual specificity protein kinase CLK4 and the RAC-gamma serine/threonine-protein kinase AKT3 have more than 2000 activity values in ChEMBL, but lack PDB structures and were so far not considered as key targets in drug development projects. Interestingly, down-regulation of AKT3 was shown to inhibit tumor growth in mouse xenograft models, providing a new treatment option for the intractable triple-negative breast cancer [27]. On the other hand, CLK4 plays a key role in controlling the function of the spliceosome and could be modulated to rectify splicing abnormalities in several diseases including cancer [28]. Due to their potential value in targeting oncogenesis [29], elucidating their 3D structures would provide a competitive advantage by guiding the rational development of novel inhibitors. Finally, highlighting under-investigated kinases can be combined with other kinome studies, e.g. druggability assessment [6], to provide directions for future drug development efforts.

## Conclusions

Annotations of the phylogenetic tree of the human kinome is an intuitive way to visualize and navigate through the continuously growing knowledge in the protein kinase field such as the number of PDB structures, compound data or kinase disease associations. The analysis options for the different data sources are manifold and include keeping track of drug development projects, repurposing clinically approved kinase inhibitors, or uncovering potential new drug targets. KinMap facilitates such analysis by providing an interactive kinome tree viewer that not only allows generating annotated images and sharing data, but also provides a user-friendly interface to explore data from different sources. The key concepts of KinMap have been described here along with two examples for using the navigation feature to search for new therapeutic indications of two known kinase inhibitors and to investigate the available structural and biochemical data on human kinases. We will continually update the built-in resources and welcome suggestions to integrate additional sources into KinMap.

## Additional file

Additional file 1: (KinMap_Examples.zip) contains the input CSV files used to generate the annotated kinome trees in Fig. 1 (Example_1_Erlotinib_NSCLC.csv), Fig. 2a (Example_2_Sunitinib_Sorafenib_Cancer.csv), and Fig. 2b (Example_3_Kinase_Stats.csv). (ZIP 5 kb)

## Abbreviations

CSV: Comma-separated values
CTTV: Center for Therapeutic Target Validation
KMAP: Native KinMap format
PDB: Protein Data Bank
PNG: Portable Network Graphics
SVG: Scalable Vector Graphics
XML: Extensible markup language
